# Optimizing the Synthetic Route of Chromone-2-carboxylic Acids: A Step forward to Speed-up the Discovery of Chromone-based Multitarget-directed Ligands

**DOI:** 10.3390/molecules24234214

**Published:** 2019-11-20

**Authors:** Fernando Cagide, Catarina Oliveira, Joana Reis, Fernanda Borges

**Affiliations:** CIQUP/Department of Chemistry and Biochemistry, Faculty of Sciences, University of Porto, Rua do Campo Alegre, 4169-007 Porto, Portugal; catarina.oliveira.fc@gmail.com (C.O.); jocostareis@gmail.com (J.R.)

**Keywords:** chromone-2-carboxylic acids, microwave-assisted reaction, multitarget ligands

## Abstract

6-Bromochromone-2-carboxylic acid (**3**) was synthesized by a microwave-assisted process. The optimization of the reaction was performed varying parameters, such as type of base/number of reagent equivalents, solvent, temperature and reaction time. The yield of the reaction was improved to 87%. The new synthetic route is versatile as several chromone-2-carboxylic acids (compounds **4B**–**10B**) were obtained with good yields (54–93%). Only in the case of the nitro substituent (compound **11B**), an ester was obtained instead of the desired carboxylic acid. Following this synthetic route chromone carboxylic acids can be attained with a high degree of purity, without the need of the tedious and expensive purification processes through column chromatography. The reaction is safe, cost-effective, fast and robust, and can be used in the development of concise and diversity-oriented libraries based on chromone scaffold. The overall study can be looked as a step forward to speed-up the discovery of chromone-based multitarget-directed ligands.

## 1. Introduction

Chromones are oxygen-containing heterocyclic compounds with a benzoannelated γ-pyrone ring widely distributed in nature and present in notable amounts in several species [1,2]. They are well-known by their diversity of pharmacological properties, such as antiallergic, anti-inflammatory, antidiabetic, antitumor, and antimicrobial [2].

**Figure 1:**
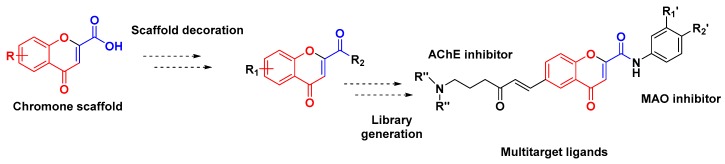
Drug design strategy followed to obtain multitarget ligands based on chromone scaffold.

Chromone (4*H*-chromen-4-one; 4*H*-1-benzopyran-4-one) is a privileged structure used in drug discovery programs, being the scaffold recognized as a pharmacophore of a great number of bioactive molecules [1,2,3]. As neurodegenerative diseases, such as Alzheimer’s and Parkinson’s disease, are characterized as multifactorial pathologies, the development of multitarget-directed ligands is a hot drug discovery issue [4]. In this context, following a multitarget strategy, our research group have developed chromone-based multitarget ligands as bifunctional acetylcholinesterase (AChE) and monoamine oxidase (MAO) inhibitors (Figure 1) [5].

Within this framework the process development and chemical route scouting for the library generation is of utmost significance. Synthetic routes must be optimized in terms of time, amount of by-products, environmental impact, yield, number of purification steps, among other parameters. In addition, the use of green and sustainable chemistry is also an important issue.

Microwave technology has been used in chemistry since the late 1970s, but it has only been implemented in organic synthesis since the mid-1980s. However, in the past few years, heating chemical reactions by microwave energy has been progressively a more popular subject [6].

The availability of safe, single-mode dedicated MW apparatus has allowed the incorporation of this technology into accelerating hit-to-lead, and lead optimization programs [7,8,9]. Their use dramatically reduces processing times, increase product yields, and enhance the purity of the product, when compared to the conventional experiments [3].

The functionalization of the chromone scaffold originates remarkable derivatives, namely those possessing a reactive carbonyl group (e.g., carboxylic acid), which are versatile synthons owing to its ability to participate in an extensive spectrum of chemical reactions [1,3,10].

**Figure 2:**
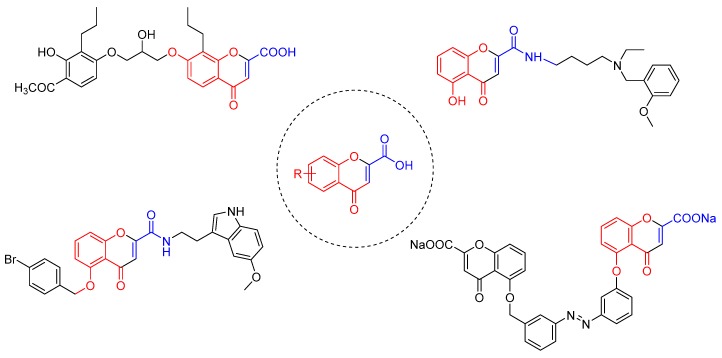
Chromones obtained from 4-oxo-4*H*-chromene-2-carboxylic acid with relevant biological activity [1,2].

In this work, our main goal was to develop an effective and fast synthetic route to obtain 4-oxo-4*H*-chromene-2-carboxylic acid and its derivatives, which are often used in medicinal chemistry to develop small libraries for structure-activity studies (Figure 2). Till now few MW reactions for the novo synthesis of this type of building blocks has been described [11,12,13].

**Scheme 1:**
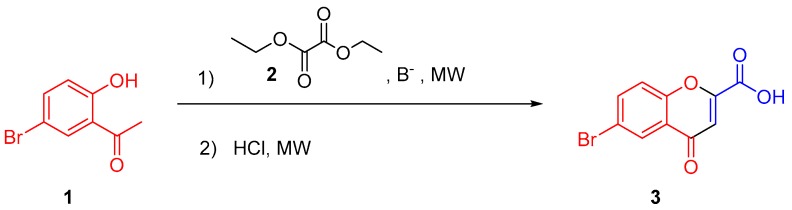
General synthetic strategy followed to obtain compound **3**.

In this context, we started the MW studies with the commercial compound 5′-bromo-2′-hydroxyacetophenone to obtain 6-bromochromone-2-carboxylic acid (**3**, Scheme 1). Although this chromone is commercially available it is very costly for drug discovery programs. After reaction optimization, the study was extended to the synthesis of other 4-oxo-4*H*-chromene-2-carboxylic acid derivatives in order to obtain the suitable synthons to speed-up the progress on the discovery of chromone-based multitarget-directed ligands.

## 2. Results and Discussion

The synthesis of 6-bromochromone-2-carboxylic acid (**3**) was initially chosen for the optimization study. As the chromone is commercially available it was used as standard allowing a rapid confirmation of its formation along the reaction by thin-layer chromatography (TLC).

The synthesis of 6-bromochromone-2-carboxylic acid (**3**) was carried out using 5′-bromo-2′-hydroxyacetophenone (**1**) as starting material and reaction conditions similar to the classic reaction described in the literature [14].

Herein we describe a method to obtain 6-bromochromone-2-carboxylic acid (**3**) by a MW-assisted synthesis (Scheme 1) comprising two steps: (1) addition of ethyl oxalate (compound **2**) and a base; (2) addition of chloridric acid (HCl) and the studies performed for its optimization, which include diverse parameters-type of base/number of reagent equivalents, solvent, temperature and reaction time.

The study was started with the conditions described in Table 1, Entry 1: sodium ethoxide (EtONa, base, 1 equiv.) in ethanol at 120 °C and 3 mL of HCl, 1 M for the hydrolysis step. The associated times of the steps (1) and (2) (Time 1 and 2, respectively) were defined as 10 min. With these conditions a low yield (14%) was obtained (Table 1, Entry 1). When the base was replaced by sodium methoxide (NaOMe) in methanol (Table 1, Entry 2) similar results were obtained. To note that these strong bases are commercially available in alcoholic solutions, and used as such. Based on the data, and our experience on the synthesis of chromones, NaOMe was the base chosen for the following studies as it is slightly cheaper, more stable and fewer side products were observed by TLC.

The increase of the equivalents number of the base (NaOMe) and of the ethyl oxalate lead to an improvement of the yield of compound **3** (Table 1, Entries 3–6), being the best reactional conditions attained with two equivalents of NaOMe and three of ethyl oxalate, with a yield of 21% (Table 1, Entry 6). After that, the effect of the acid concentration used in the step 2) was studied (Table 1, Entries 7–8). Although a change of 1 M to 6 M was performed, the yield up only to 30% (Table 1, Entry 8).

Additional modifications in the temperature and time of the reaction were performed. The increase of the temperature of the first reaction step to 140 °C resulted in a decrease of the yield to 19% (Table 1, Entry 9). However, keeping the temperature at 120 °C and increasing the reaction time of the step (1) to 20 min, a small increment of the yield to 34% was observed (Table 1, Entry 10). Nevertheless, longer times of reaction (30 min) did not produce any performance upsurge (Table 1, Entry 11).

To improve the acetophenone solubilization the solvent of the reaction was also changed to a less polar (Table 1, Entries 12,13). The most significant results were obtained when dioxane was used with an increase in yield going from 34% to 68% (Table 1, Entry 13).

The final optimization step was related with the increment of the hydrolysis time of the ester (step 2, Table 1, Entries 14,15): 40 min allowed to obtain compound 3 with a yield of 87% (Table 1, Entry 15). Likewise, longer times of reaction (50 min) did not produce an additional increase of the performance of the reaction (Table 1, Entry 16).

After the reaction optimization its versatility was verified using dissimilar 2′-hydroxyacetophenone derivatives (**A**) to obtain chromone-2-carboxylic acid derivatives (**B**) having different type of substituents (Table 2). The selected substituents are often used in medicinal chemistry programs [15,16].

When the 2′-hydroxyacetophenone without substituents in the aromatic ring (compound **4A**) was used, a decrease in yield (compound **4B**, 54%, Table 2) was observed in comparison with the obtained 6-bromochromone-2-carboxylic acid (compound **3**, 87%, Table 1, Entry 15). The replacement of bromine by a chlorine in the aromatic ring (compound **5A**) allowed to obtain the desired chromone with a slight lower yield (compound **5B**, 71%, Table 2). A similar behavior was observed when the 2′-hydroxyacetophenone substituent was a methyl group (compound **6B**, 64%, Table 2). When the acetophenone is substituted with one methoxyl group (compounds **7A**, **8A** and **9A**) a similar reaction performance to compound **3**, with yields between 81–93% (compounds **7B**, **8B** and **9B**, Table 2) was obtained. However, when the 2′-hydroxyacetophenone has two methoxy substituents in positions 7 and 8 (compound **10A**) a reduction of the reaction yield to 62% was observed (compound **10B**, Table 2).

As all the purification steps were based only on the washing process, without further purification by column chromatography, a loss of mass along the process was, in general, observed.

Finally, when the aromatic ring possesses a nitro group in position 6 (compound **11A**), instead of the desired chromone carboxylic acid, the corresponding methyl ester was obtained. This shortcoming can be related to the presence of a highly deactivating substituent that can preclude ester hydrolysis by stabilizing the reaction intermediate or by bring about a compound with low solubility in the reaction media.

## 3. Material and Methods

### 3.1. Apparatus

Microwave-assisted synthesis was performed in a Biotage^®^ Initiator Microwave Synthesizer (Biotage, Uppsala, Sweden). ^1^H- and ^13^C-NMR spectra were recorded on an AMX 400 NMR spectrometer (Bruker, Billerica, MA, USA). Spectra were recorded at room temperature in 5 mm outside diameter (o.d.) tubes. Tetramethylsilane (TMS) was used as internal standard, chemical shifts are expressed in ppm (δ) and *J* in Hz. For the DEPT sequence, the width of the 90° pulse for ^13^C was 4 μs, and that of the 90° pulse for ^1^H was 9.5 μs; the delay 2JC,H −1 was set to 3.5 ms (underlined values). The melting point were carried out on a Stuart Scientific Melting Point SMP1 apparatus (Stuart Scientific, Staffordshire, UK).

### 3.2. Reagents and General Conditions

All reagents were purchased from Sigma-Aldrich Química S.A. (Lisbon, Portugal) and TCI Chemicals Europe (Paris, French). All solvents were pro analysis grade from Merck (Darmstadt, Germany), Carlo Erba Reagents (Val de Reuil, France) and Scharlab (Barcelona, Spain).

Thin layer chromatography (TLC) was performed on precoated silica gel 60 F254 acquired from Merck with layer thickness of 0.2 mm. Reaction control was monitored using ethyl acetate and/or ethyl acetate:methanol (9:1) and spots were visualized under UV detection at 254 and 366 nm.

### 3.3. Synthesis of 4-Oxo-4H-chromene-2-carboxylic acids ***3***–***11***

#### General Procedure

The appropriate hydroxyacetophenone (**1** or **A**, 1.16 mmol) was dissolved in dioxane (2 mL) in a MW vial. Then diethyl oxalate (3.49 mmol, 474 µL) and a solution of MeONa in MeOH (2.32 mmol, 531 µL, 25% *w*/*w*) were added. The resulting solution was heated to 120 °C for 20 min. Then, a solution of HCl (18 mmol, 3 mL, 6 M) was added and the reaction was heated to 120 °C for 40 min. The reaction was decanted over water (50 mL) and the solid formed was filtered and washed with water. The solid was then dried, washed with dichloromethane and dried again.

*6-Bromo-4-oxo-4H-chromene-2-carboxylic acid* (**3**): η = 87%, ^1^H-NMR (DMSO-d_6_) δ: 8.12 (1H, *d*, *J* = 2.4 Hz, H5), 8.03 (1H, *dd*, *J*_1_ = 9.0 Hz, *J*_2_ = 2.5 Hz, H7), 7.74 (1H, *d*, *J* = 9.0 Hz,), 6.95 (1H, *s*, H3). ^13^C-NMR (DMSO-d_6_) δ 176.4 (C4), 160.9 (COOH), 154.4 (C2), 153.6 (C8a), 137.7 (C6), 126.9 (C5), 125.2 (C4a), 121.5 (C3), 118.4 (C6), 113.4 (C8). M.p.: 263–267 °C.

*4-Oxo-4H-chromene-2-carboxylic acid* (**4B**): η = 54%, ^1^H-NMR (MeOD) δ: 8.17 (1H, *dd*, *J*_1_ = 8.0 Hz, *J*_2_ = 1.7 Hz, H5), 7.88 (1H, *ddd*, *J*_1_ = 8.7 Hz, *J*_2_ = 7.1 Hz, *J*_3_ = 1.7 Hz, H7), 7.72 (1H, *dd*, *J*_1_ = 8.7, *J*_1_ = 1.1 Hz, H8), 7.55 (1H, *ddd*, *J*_1_ = 8.0 Hz, *J*_2_ = 7.1 Hz, *J*_3_ = 1.1 Hz, H6), 7.08 (1H, *s*, H3). ^13^C-NMR (MeOD-d_4_) δ:179.4 (C4), 161.5 (COOH), 156.2 (C2), 153.9 (C8a), 135.1 (C7), 125.9 (C5), 124.9 (C6), 123.8 (C4a), 118.6 (C3), 113.3 (C8). M.p.: >285 °C dec.

*6-Chloro-4-oxo-4H-chromene-2-carboxylic acid* (**5B**): η = 71%, ^1^H-NMR (DMSO-d_6_) δ: 8.08 (1H, *d*, *J* = 2.6 Hz, H5), 7.83 (1H, *dd*, *J*_1_ = 9.0, *J*_2_ = 2.6 Hz, H7), 7.71 (1H, *d*, *J* = 9.0 Hz, H8), 7.06 (1H, *s*, H3). ^13^C-NMR (DMSO-d_6_) δ: 176.6 (C4), 161.2 (COOH), 154.1 (C2), 153.6 (C8a), 135.0 (C7), 130.5 (C6), 124.8 (C5), 123.9 (C2a), 121.4 (C3), 113.3 (C8). M.p.: 280–284 °C.

*6-Methyl-4-oxo-4H-chromene-2-carboxylic acid* (**6B**): η = 64%, ^1^H-NMR (400 MHz, MeOD) δ: 7.92 (1H, *d*, *J* = 2.3 Hz, H5), 7.68 (1H, *dd*, *J*_1_ = 8.7, *J*_2_ = 2.3 Hz, H6), 7.58 (1H, *d*, *J* = 8.6 Hz, H8), 7.02 (1H, *s*, H3), 2.48 (3H, *s*, CH_3_). ^13^C-NMR (MeOD-d_4_) δ: 180.8 (C4), 163.0 (COOH), 156.0 (C2), 155.1 (C8a), 137.8 (C7), 137.7 (C6), 125.5 (C5), 125.0 (C4a), 119.8 (C3), 114.6 (C8), 21.0 (CH_3_). M.p.: >279 °C dec.

*7-Methoxy-4-oxo-4H-chromene-2-carboxylic acid* (**7B**): η = 83%, ^1^H-NMR (DMSO-d_6_) δ: 7.95 (1H, *d*, *J* = 9.2 Hz, H5), 7.22 (1H, *d*, *J* = 2.4 Hz, H8), 7.12 (1H, *dd*, *J*_1_ = 9.2 Hz, *J*_2_ = 2.4 Hz, H6), 6.86 (1H, *s*, H3), 3.94 (3H, *s*, OCH_3_). ^13^C-NMR (DMSO-d_6_) δ: 177.2 (C4), 165.1 (C7), 161.9 (COOH), 158.0 (C2), 153.6 (C8a), 126.9 (C5), 118.2 (C4a), 116.1 (C3), 114.2 (C6), 101.5 (C8), 56.8 (OCH_3_). M.p.: 283–287 °C.

*6-Methoxy-4-oxo-4H-chromene-2-carboxylic acid* (**8B**): η = 81%, ^1^H-NMR (DMSO-d_6_) δ: 7.71 (1H, *d*, *J* = 9.2 Hz, H8), 7.47 (1H, *dd*, *J*_1_ = 9.2 Hz, *J*_1_ = 3.2 Hz, H7), 7.42 (1H, *d*, *J* = 3.2 Hz, H5), 6.89 (1H, *s*, H3), 3.88 (3H, *s*, OCH_3_). ^13^C-NMR (DMSO-d_6_) δ: 177.3 (C4), 161.5 (COOH), 157.0 (C2), 153.0 (C6), 150.2 (C8a), 124.5 (C4a), 124.4 (C7), 120.5 (C3), 112.5 (C8), 104.5 (C5), 55.8 (OCH_3_). M.p.: 293–294 °C.

*5-Methoxy-4-oxo-4H-chromene-2-carboxylic acid* (**9B**): η = 93%, ^1^H-NMR (MeOD) δ: 7.73 (1H, *dd*, *J*_1_ = 8.5 Hz, *J*_2_ = 8.5 Hz, 7H), 7.20 (1H, *dd*, *J* = 8.5, *J*_2_ = 0.9 Hz, H8), 7.03 (1H, *dd*, *J* = 8.5, *J*_2_ = 0.9 Hz, H6), 6.91 (1H, *s*, H3), 3.95 (3H, *s*, OCH_3_). ^13^C-NMR (MeOD-d_4_) δ: 179.1 (C4), 161.4 (COOH), 159.6 (C2), 158.1 (C5), 151.8 (C8a), 135.4 (C7) 115.0 (C3), 114.2 (C4a), 110.1 (C6), 107.0 (C8), 55.3 (OCH_3_). M.p.:> 256°C dec.

*7,8-Dimethoxy-4-oxo-4H-chromene-2-carboxylic acid* (**10B**): η = 62%, ^1^H-NMR (DMSO-d_6_) δ: 7.77 (1H, *d*, *J* = 9.0 Hz, H5), 7.31 (1H, *d*, *J* = 9.0 Hz, H6), 6.82 (1H, *s*, H3), 3.95 (3H, *s*, OCH_3_), 3.91 (3H, *s*, OCH_3_). ^13^C-NMR (DMSO-d_6_) δ: 176.9 (C4), 161.4 (COOH), 156.8 (C2), 152.9 (C7), 149.7 (C8a), 136.5 (C8), 120.1 (C3), 118.3 (C4a), 112.9 (C5), 111.4 (C7), 60.9 (OCH_3_), 56.5 (OCH_3_). M.p.: >287 °C dec.

*Methyl 6-nitro-4-oxo-4H-chromene-2-carboxylate* (**11B**): η = 83%, ^1^H-NMR (DMSO-d_6_) δ: 8.72 (1H, *d*, *J* = 2.8 Hz, H6), 8.61 (1H, *dd*, *J*_1_ = 9.2, *J*_2_ = 2.8 Hz, H7), 8.00 (1H, *d*, *J* = 9.2 Hz, H7), 7.03 (1H, *s*, H3), 3.57 (3H, *s*, OCH_3_). ^13^C-NMR (DMSO-d_6_) δ: 176.9 (C4), 160.9 (COOCH_3_), 158.5 (C2), 154.0 (C8a), 144.7 (C6), 129.2 (C7), 123.7 (C4a), 121.2 (C5), 120.9 (C3), 113.7 (C8), 66.3 (OCH_3_). M.p.: 274–279 °C.

Details of apparatus and NMR spectra of the synthesized compounds are available in Supplementary Materials.

## 4. Conclusions

The synthesis of 6-bromochromone-2-carboxylic acid (**3**) using 5′-bromo-2′-hydroxy-acetophenone (**1**) as starting material and ethyl oxalate (**2**) was successfully accomplished using a MW-assisted process. For the optimization process a number of parameters were adjusted, such as type of base/number of reagent equivalents, solvent, temperature and reaction time. The yield of the reaction was improved to 87%.

The new synthetic route is versatile as several chromone-2-carboxylic acids (compounds **4B–10B**) were obtained with good yields (54–93%). Only in the case of the nitro substituent (compound **11B**), an ester was obtained instead of the desired carboxylic acid.

Following this synthetic route, a variety of chromone carboxylic acids can be attained with a high degree of purity, without the need of the tedious and expensive purification processes through column chromatography. This is a great advantage for the scale-up of the process.

The reaction is safe, cost-effective, fast, robust, and can be used in the development of concise and diversity-oriented libraries based on chromone scaffold. The overall study can be looked as a step forward to speed-up the progress of chromone-based multitarget-directed ligands.

## Figures and Tables

**Table 1:** Microwave-assisted optimization conditions for the synthesis of compound **3**.

Entry	Base (Equiv)	2 (Equiv)	Solvent	T (°C)	Time 1 * (min)	Acid (V = 3 mL)	Time 2 * (min)	Yield (%)
**1**	EtONa (1)	1	EtOH	120	10	HCl (1 M)	10	14
**2**	MeONa (1)	1	MeOH	120	10	HCl (1 M)	10	15
**3**	MeONa (2)	1	MeOH	120	10	HCl (1 M)	10	19
**4**	MeONa (3)	1	MeOH	120	10	HCl (1 M)	10	17
**5**	MeONa (2)	2	MeOH	120	10	HCl (1 M)	10	19
**6**	MeONa (2)	3	MeOH	120	10	HCl (1 M)	10	21
**7**	MeONa (2)	3	MeOH	120	10	HCl (3 M)	10	23
**8**	MeONa (2)	3	MeOH	120	10	HCl (6 M)	10	30
**9**	MeONa (2)	3	MeOH	140	10	HCl (6 M)	10	19
**10**	MeONa (2)	3	MeOH	120	20	HCl (6 M)	10	34
**11**	MeONa (2)	3	MeOH	120	30	HCl (6 M)	10	34
**12**	MeONa (2)	3	^i^PrOH	120	20	HCl (6 M)	10	51
**13**	MeONa (2)	3	Dioxane	120	20	HCl (6 M)	10	68
**14**	MeONa (2)	3	Dioxane	120	20	HCl (6 M)	30	81
**15**	MeONa (2)	3	Dioxane	120	20	HCl (6 M)	40	87
**16**	MeONa (2)	3	Dioxane	120	20	HCl (1 M)	50	85

* The temperature was maintained for the two reaction steps (1) and (2) (Time 1 and 2, respectively).

**Table 2:**
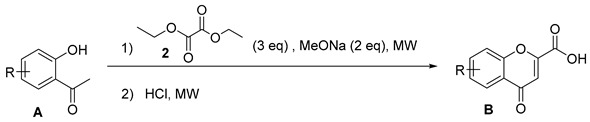
Synthesis of chromone-2-carboxylic acids by microwave-assisted reaction.

Compound	2′-Hydroxyacetophenone (A)	Chromone-2-Carboxylic Acid (B)	Yield (%)
**4**	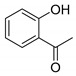	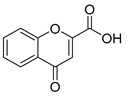	54 ^a^
**5**	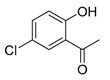	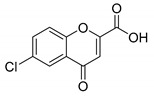	71
**6**	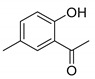	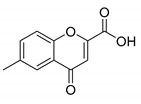	64
**7**	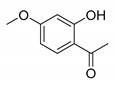	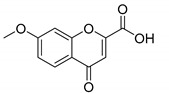	**83**
**8**	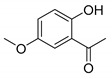	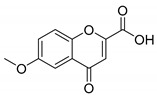	**81**
**9**	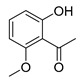	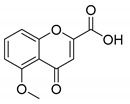	93
**10**	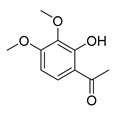	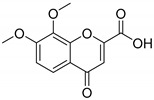	62
**11**	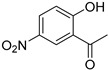	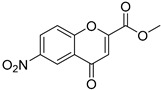	83

^a^. The yield of the compound has been previously described, for similar reaction conditions and with a domestic MW apparatus, as 93% [17].

## Supplementary Materials

The following are available online, Details apparatus and NMR spectra of the synthesized compounds can be found in the Supporting Information.

## Abbreviations

AChE	Acetylcholinesterase
MAO	Monoamine oxidase
MW	Microwave
TLC	Thin-layer chromatography
