# Supplementary material for: Optimizing the Synthetic Route of Chromone-2-carboxylic Acids: A Step forward to Speed-up the Discovery of Chromone-based Multitarget-directed Ligands
Source: Molecules. 2019 Nov 20;24(23):4214. doi: 10.3390/molecules24234214 (PMC6930484; doi:10.3390/molecules24234214)
Supplement: Supplementary file 1 [file molecules-24-04214-s001.pdf]

Supplementary information

Optimizing the synthetic route of chromone-2-carboxylic acids: a step forward to speed-up the discovery of chromone-based multitarget-directed ligands

Fernando Cagide[1*], Catarina Oliveira[1], Joana Reis[1#], Fernanda Borges[1]*

[1]CIQUP/Department of Chemistry and Biochemistry, Faculty of Sciences, University of Porto, Rua do Campo Alegre, 4169-007, Porto, Portugal;

S.1 Apparatus

Microwave-assisted synthesis was performed in a Biotage® Initiator Microwave Synthesizer.

NMR was recorded on a Bruker AMX 400 NMR spectrometer. ^{1}H and ^{13}C NMR spectra of samples were recorded at room temperature in 5 mm outside diameter (o.d.) tubes. Tetramethylsilane (TMS) was used as internal standard, chemical shifts are expressed in ppm (δ) and J in Hz. For the DEPT sequence, the width of the 90◦ pulse for ^{13}C was 4 μs, and that of the 90º pulse for ^{1}H was 9.5 μs; the delay $2J_{C,H}{}^{-1}$ was set to 3.5 ms (underlined values).

The melting point were carried out on a Stuart Scientific Melting Point SMP1 (United Kingdom)

S.2Reagents and general conditions

All reagents were purchased from Sigma-Aldrich Química S. A. and TCI Chemicals. All solvents were *pro analysis* grade from Merck, Carlo Erba Reagents and Scharlab.

Thin layer chromatography (TLC) was performed on precoated silica gel 60 F254 acquired from Merck with layer thickness of 0.2 mm. Reaction control was monitored using ethyl acetate and/or ethyl acetate:methanol (9:1) and spots were visualized under UV detection at 254 and 366 nm.

S.3 NMR spectra of the compound (3,4B-11B)

- 6-Bromo-4-oxo-4*H*-chromene-2-carboxylic acid (3)

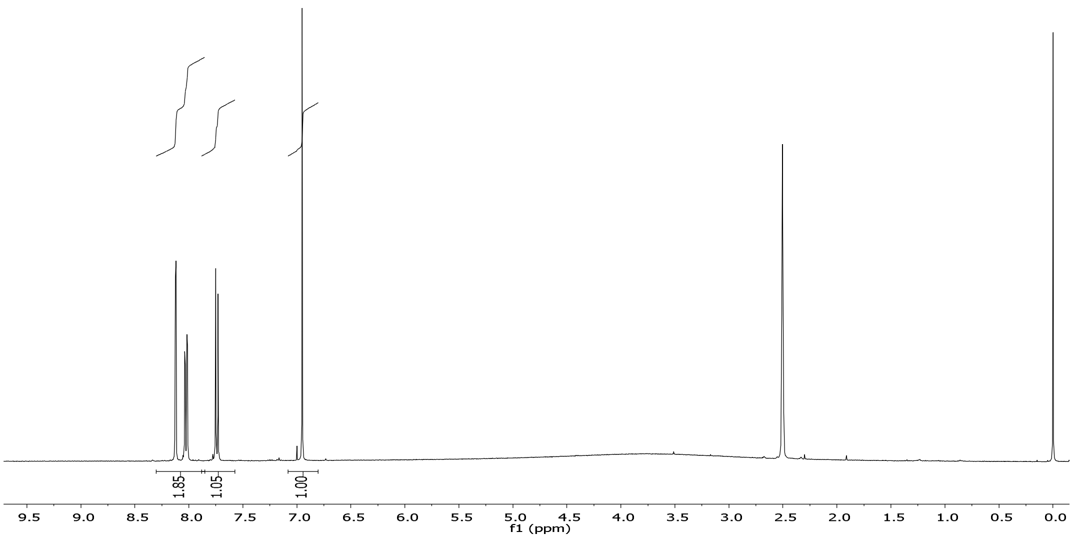

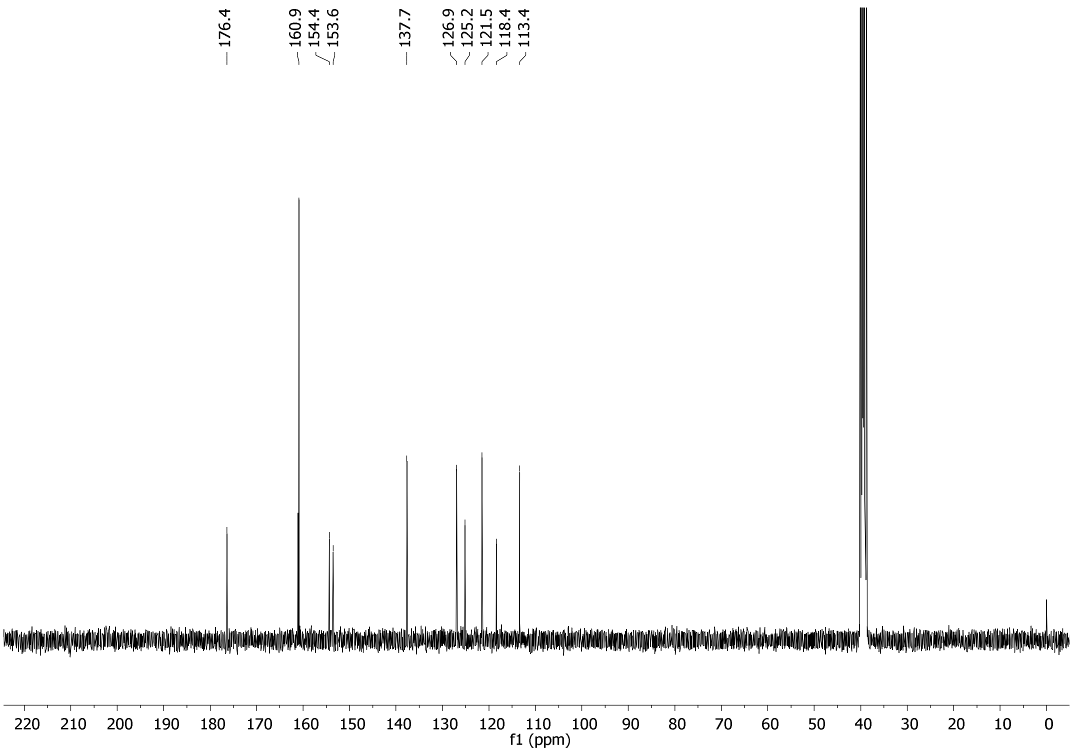

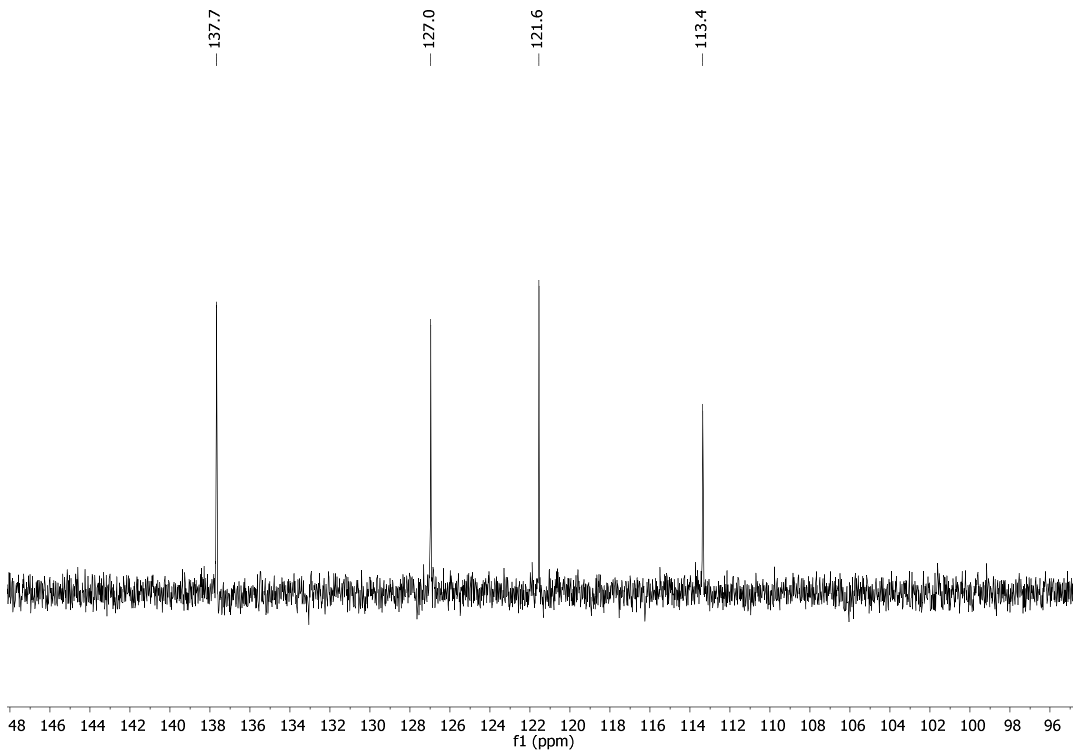

- 4-Oxo-4*H*-chromene-2-carboxylic acid (4B)

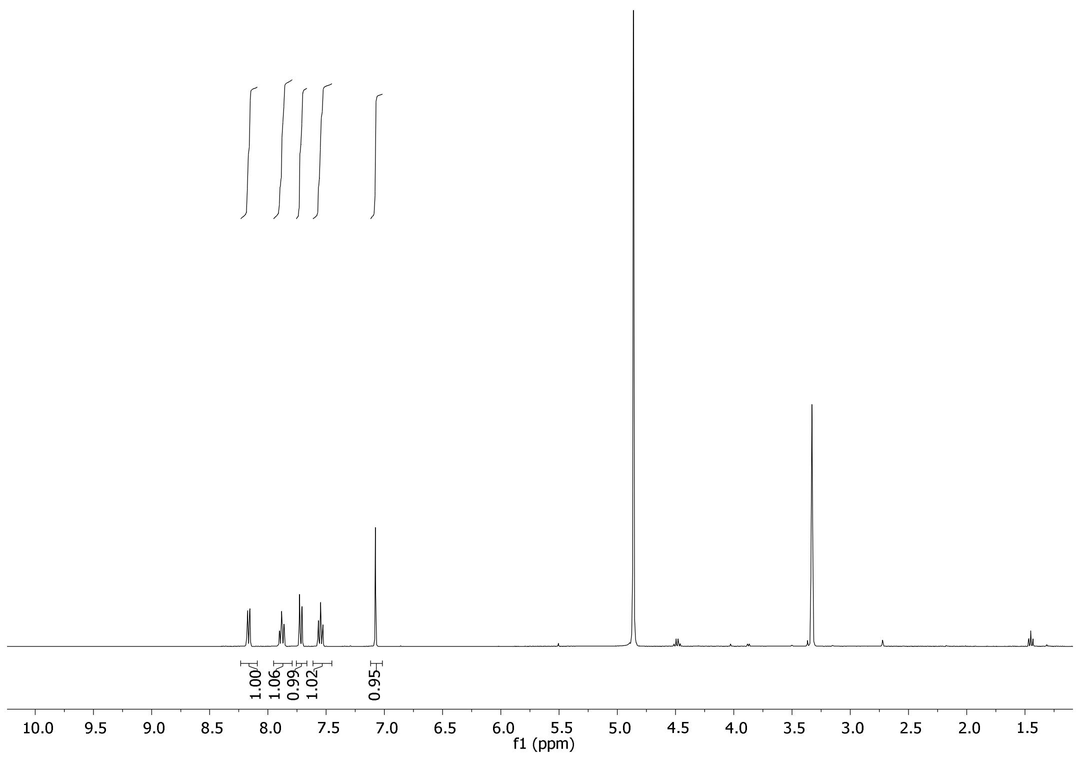

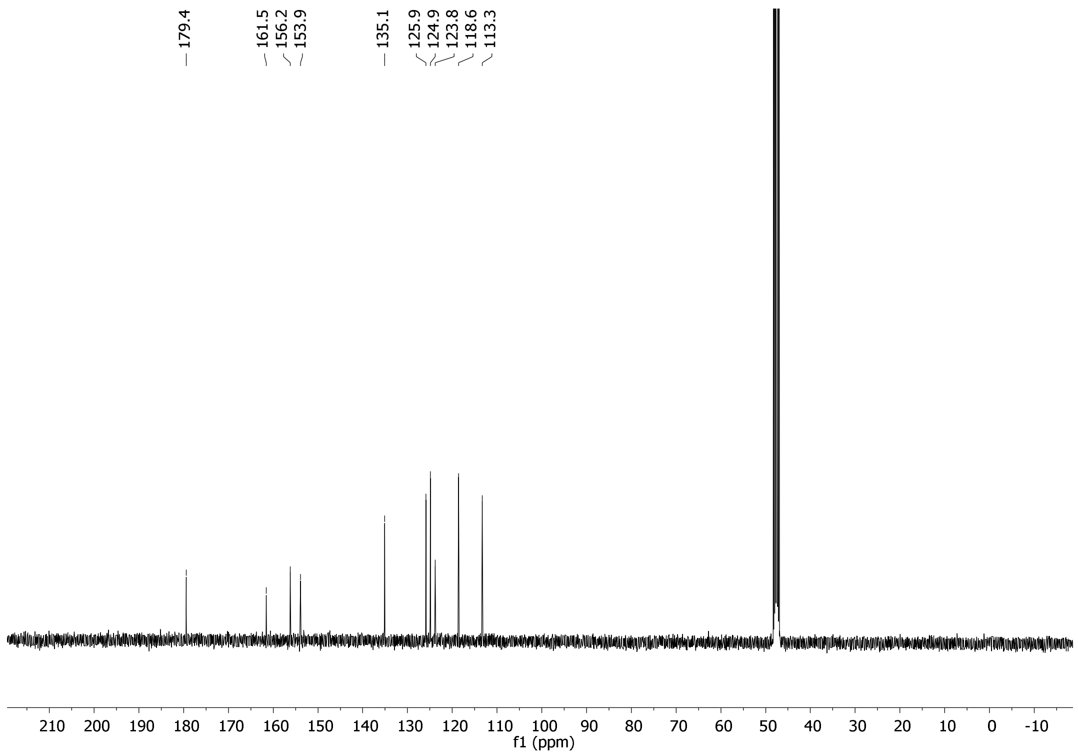
179.4
161.5
156.2
153.9
135.1
125.9
124.9
123.8
118.6
113.3
210 200 190 180 170 160 150 140 130 120 110 100 90 80 70 60 50 40 30 20 10 0 -10
f1 (ppm)

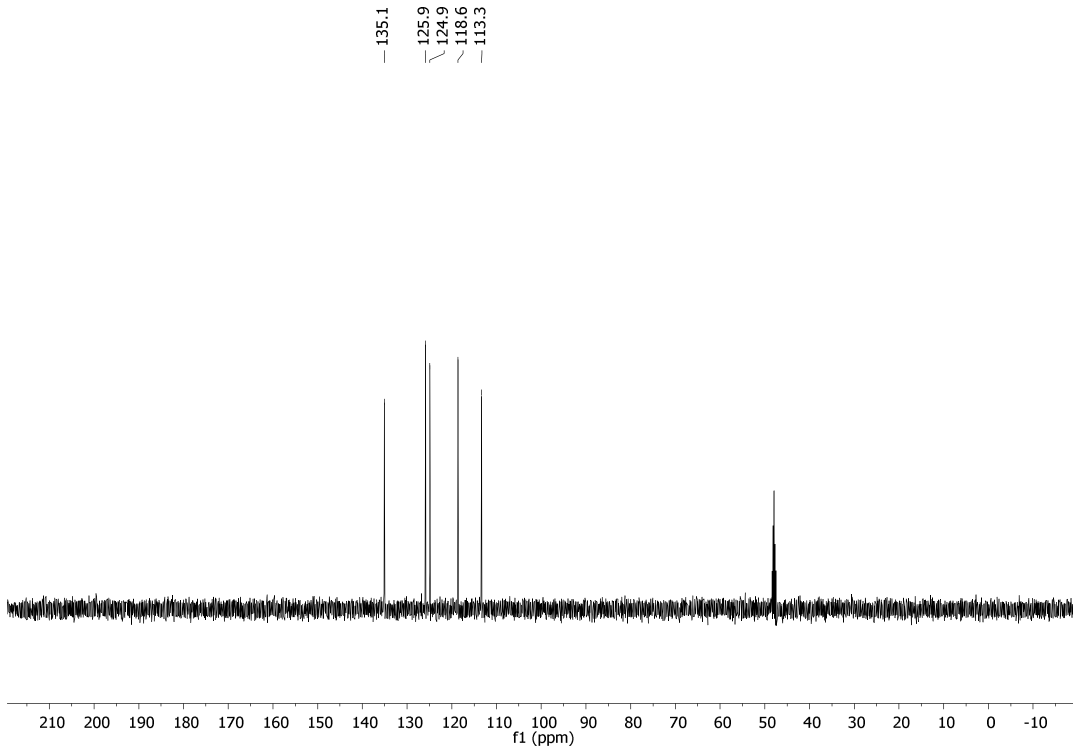
135.1
125.9
124.9
118.6
113.3
210 200 190 180 170 160 150 140 130 120 110 100 90 80 70 60 50 40 30 20 10 0 -10
f1 (ppm)

- **6-Chloro-4-oxo-4*H*-chromene-2-carboxylic acid (5B)**

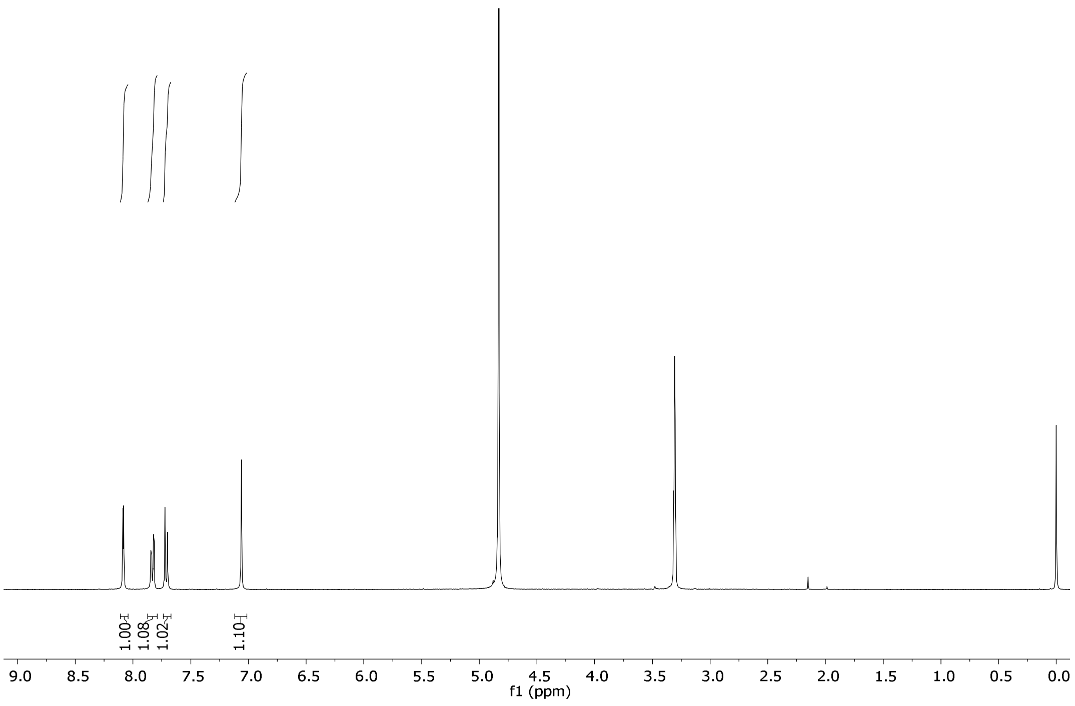

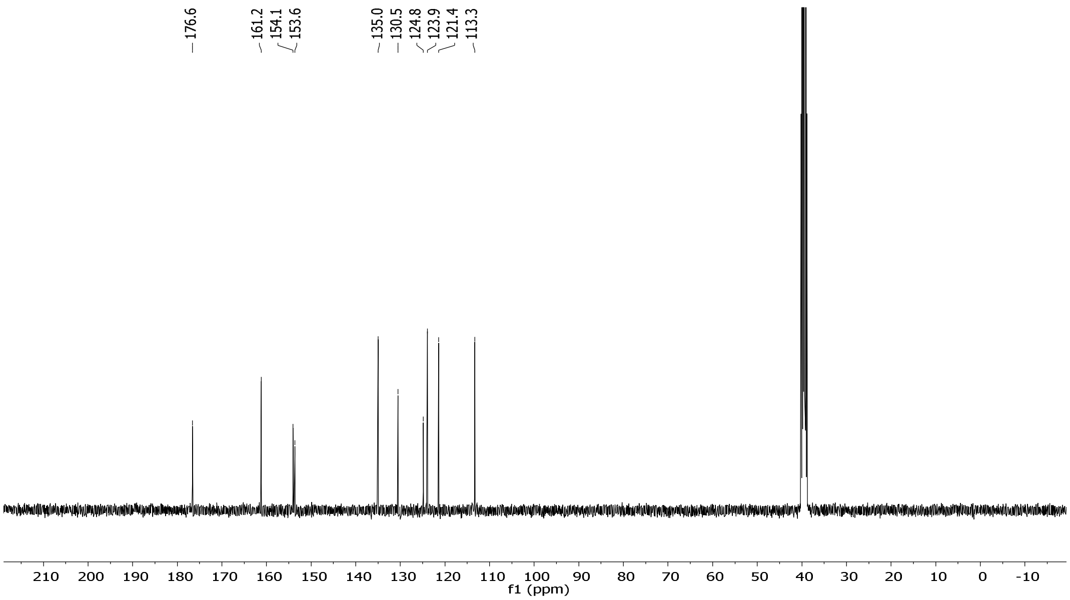

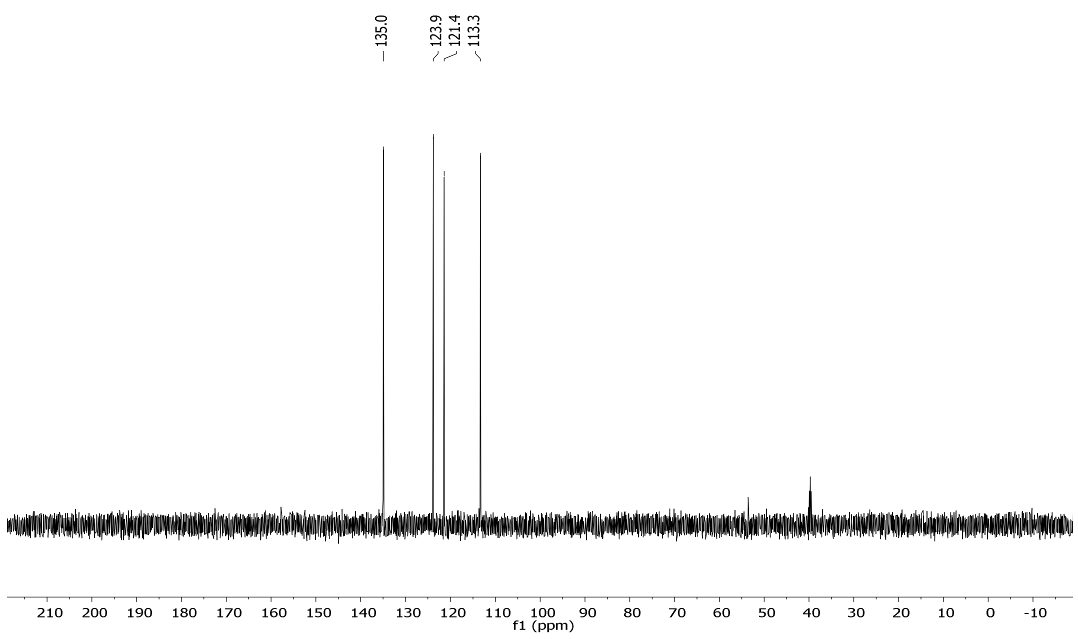

- 6-Methyl-4-oxo-4*H*-chromene-2-carboxylic acid (6B)

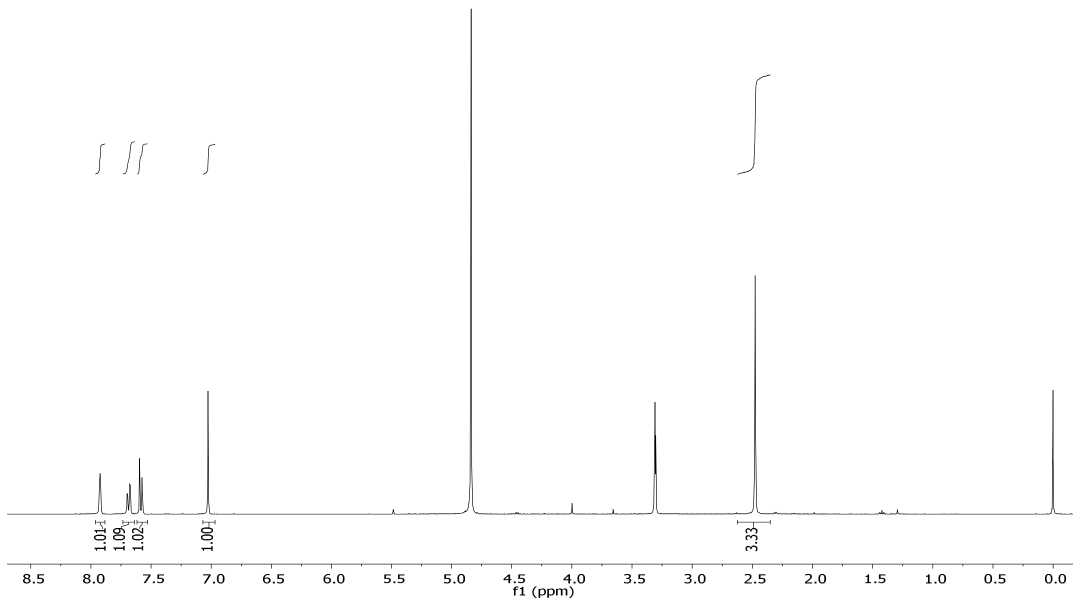

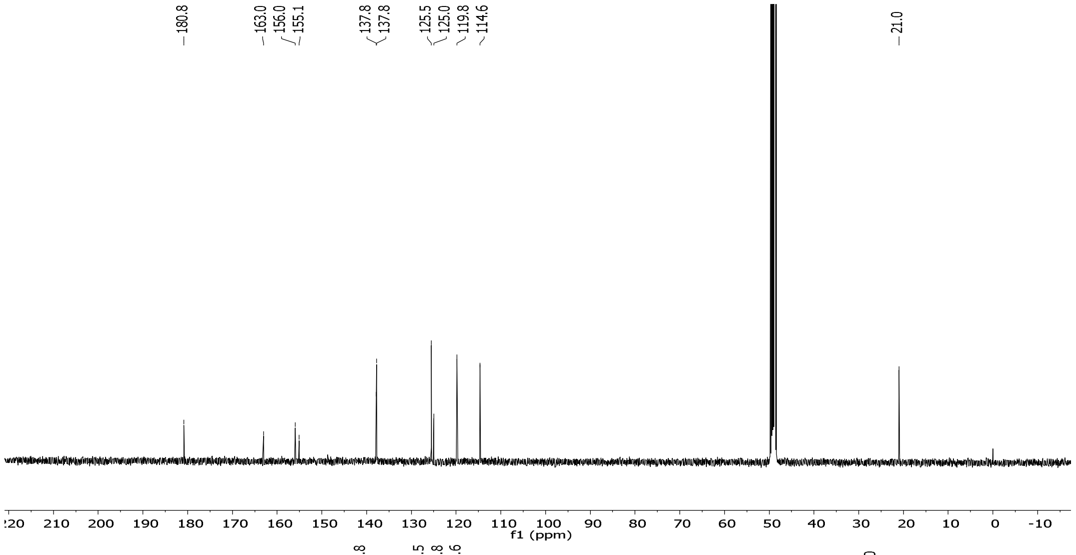
180.8
163.0
156.0
155.1
137.8
137.8
125.5
125.0
119.8
114.6
21.0
220 210 200 190 180 170 160 150 140 130 120 110 100 90 80 70 60 50 40 30 20 10 0 -10
f1 (ppm)

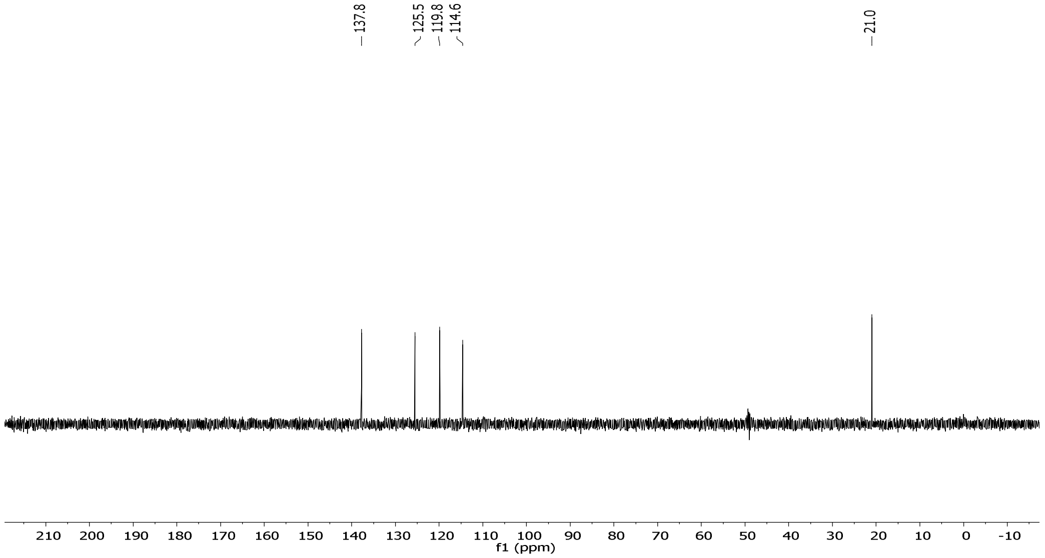
137.8
125.5
119.8
114.6
21.0
210 200 190 180 170 160 150 140 130 120 110 100 90 80 70 60 50 40 30 20 10 0 -10
f1 (ppm)

- **7-Methoxy-4-oxo-4*H*-chromene-2-carboxylic acid (7B)**

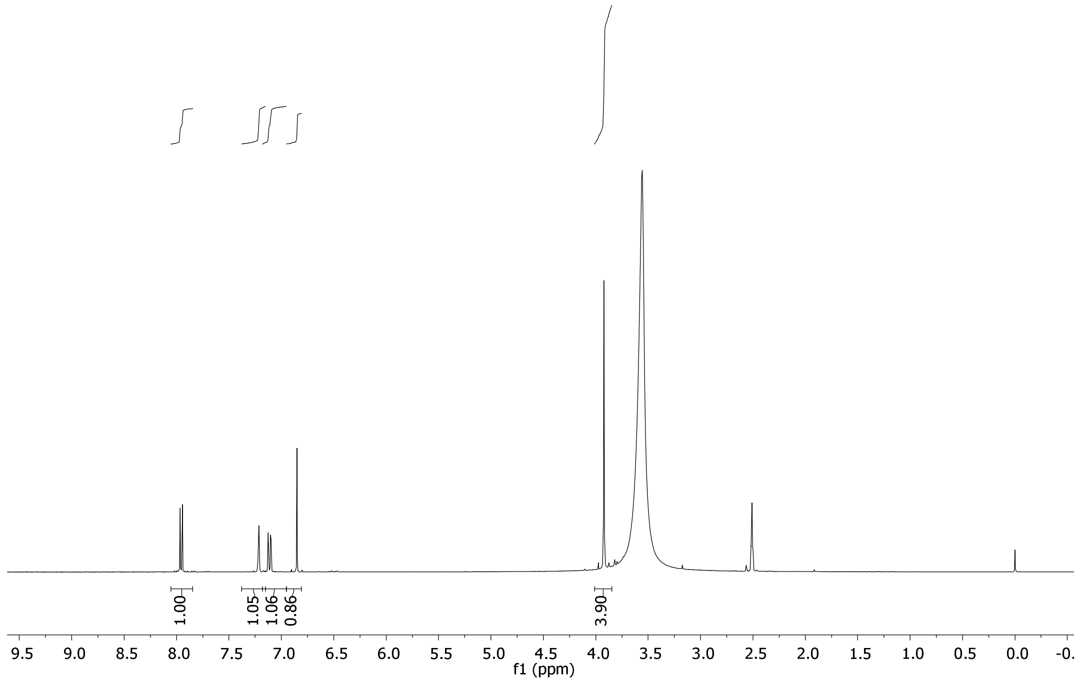

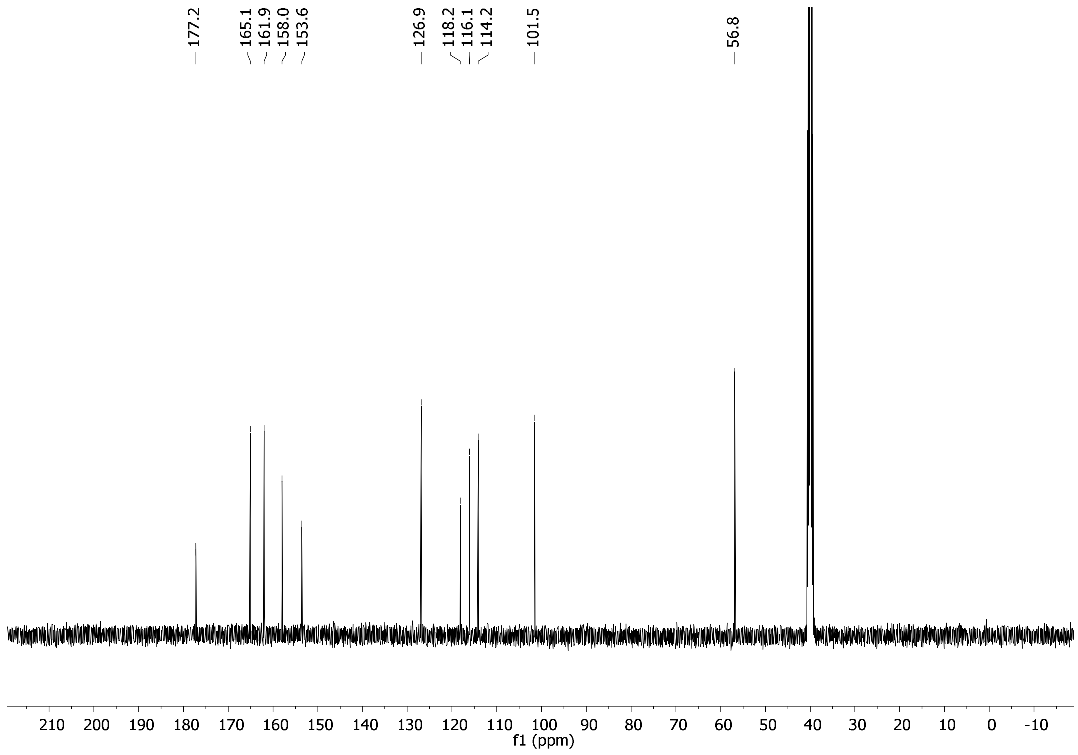

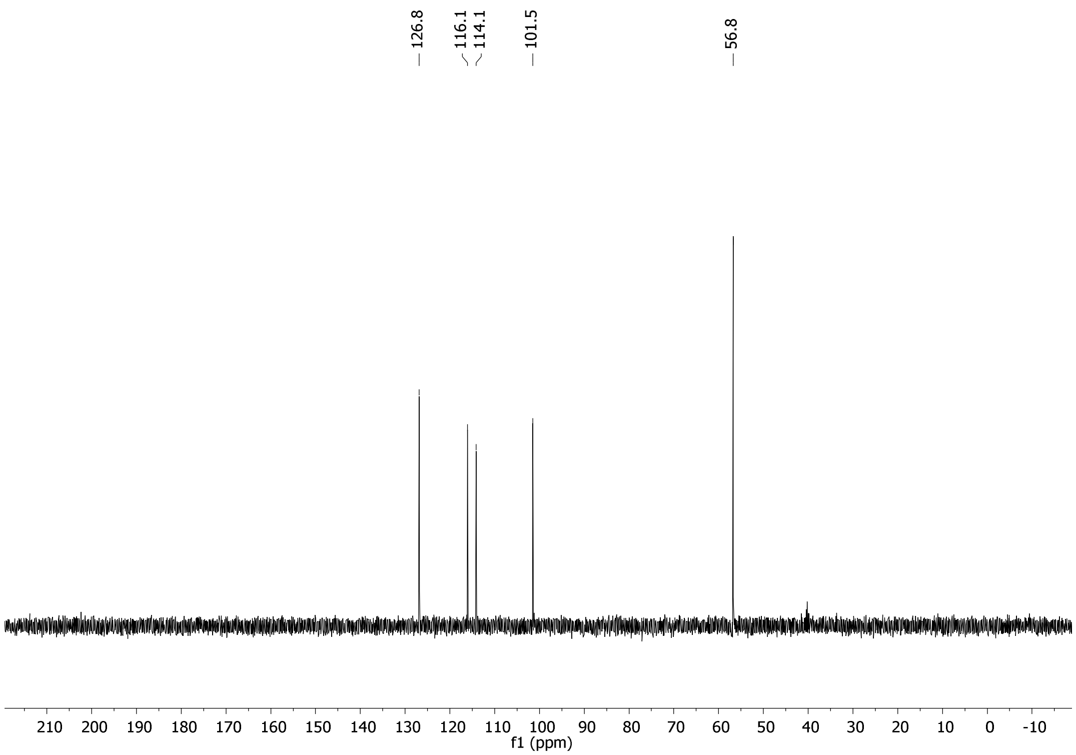

- 6-Methoxy-4-oxo-4*H*-chromene-2-carboxylic acid (8B):

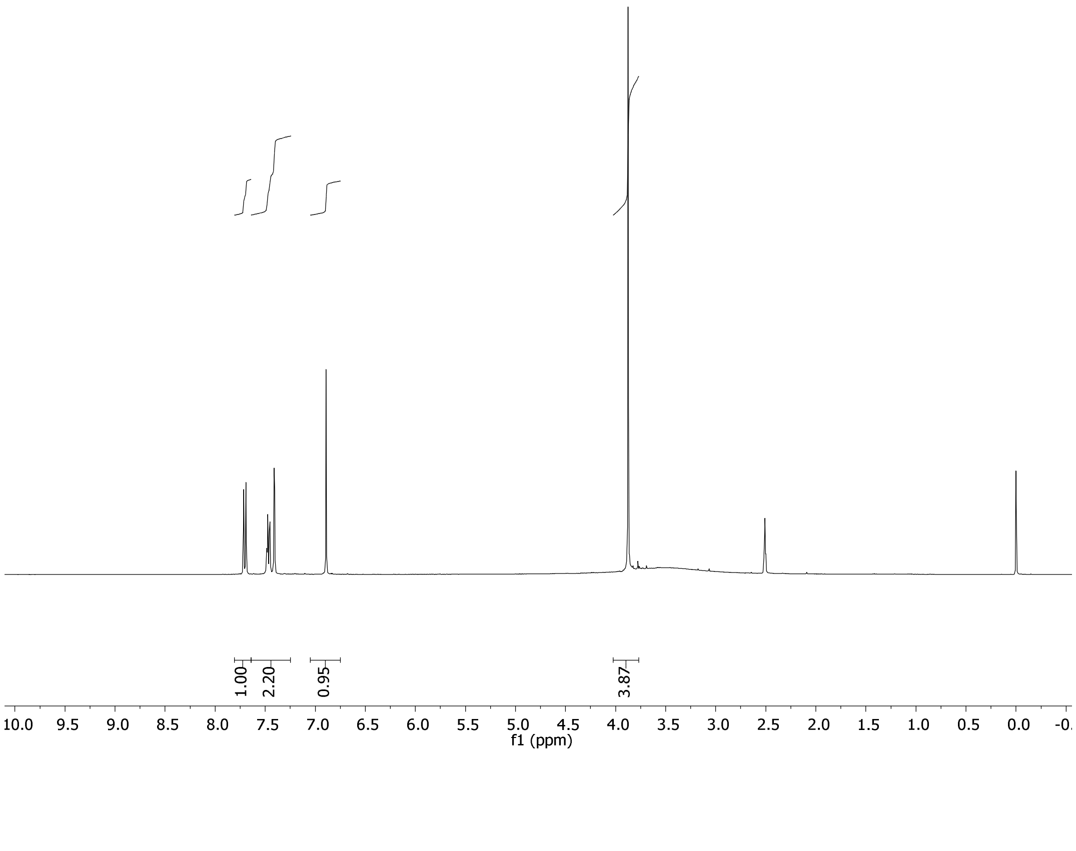

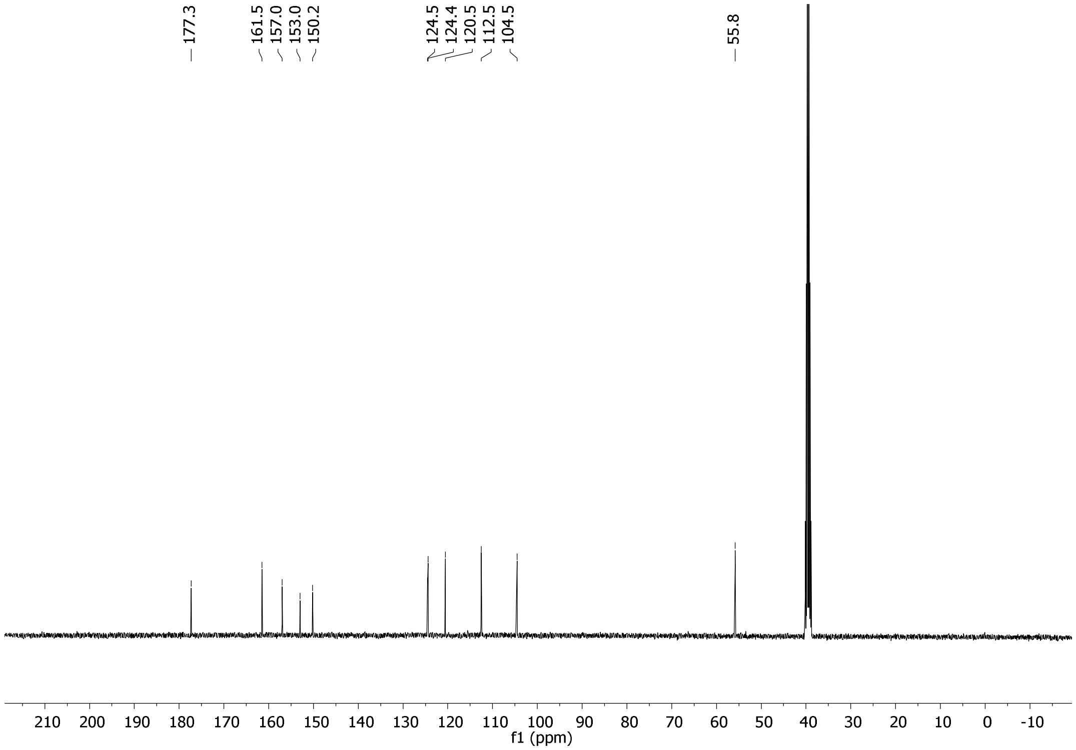
177.3
161.5
157.0
153.0
150.2
124.5
124.4
120.5
112.5
104.5
55.8
210 200 190 180 170 160 150 140 130 120 110 100 90 80 70 60 50 40 30 20 10 0 -10
f1 (ppm)

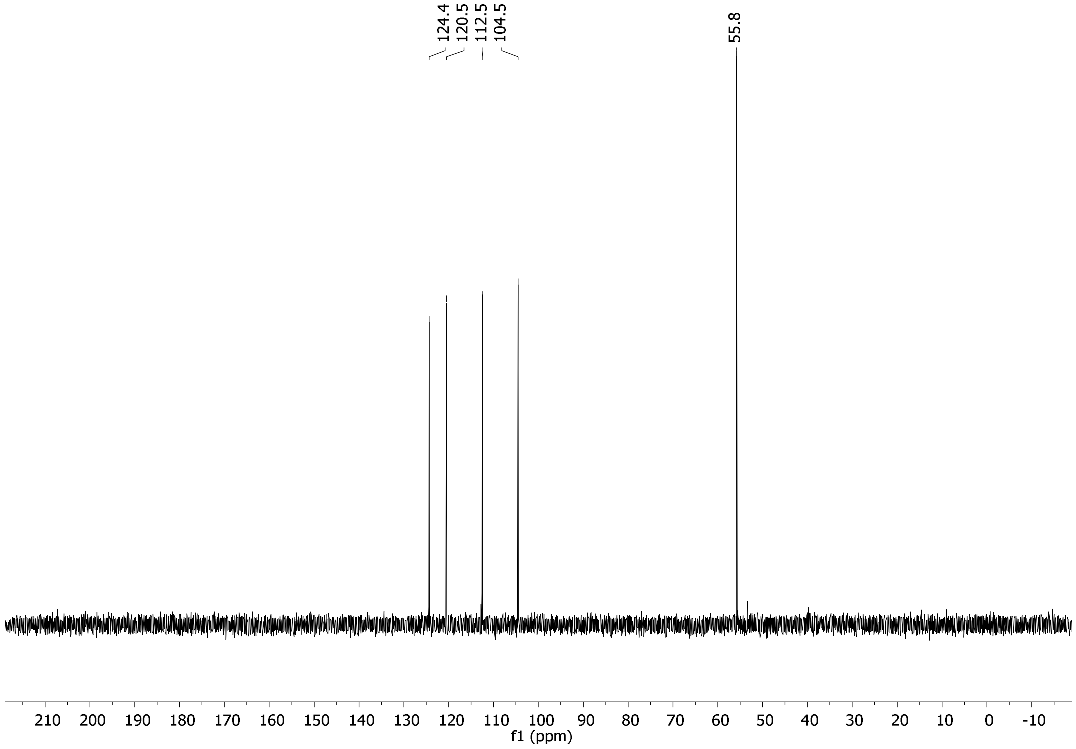
124.4
120.5
112.5
104.5
55.8
210 200 190 180 170 160 150 140 130 120 110 100 90 80 70 60 50 40 30 20 10 0 -10
f1 (ppm)

- 5-Methoxy-4-oxo-4*H*-chromene-2-carboxylic acid (9B)

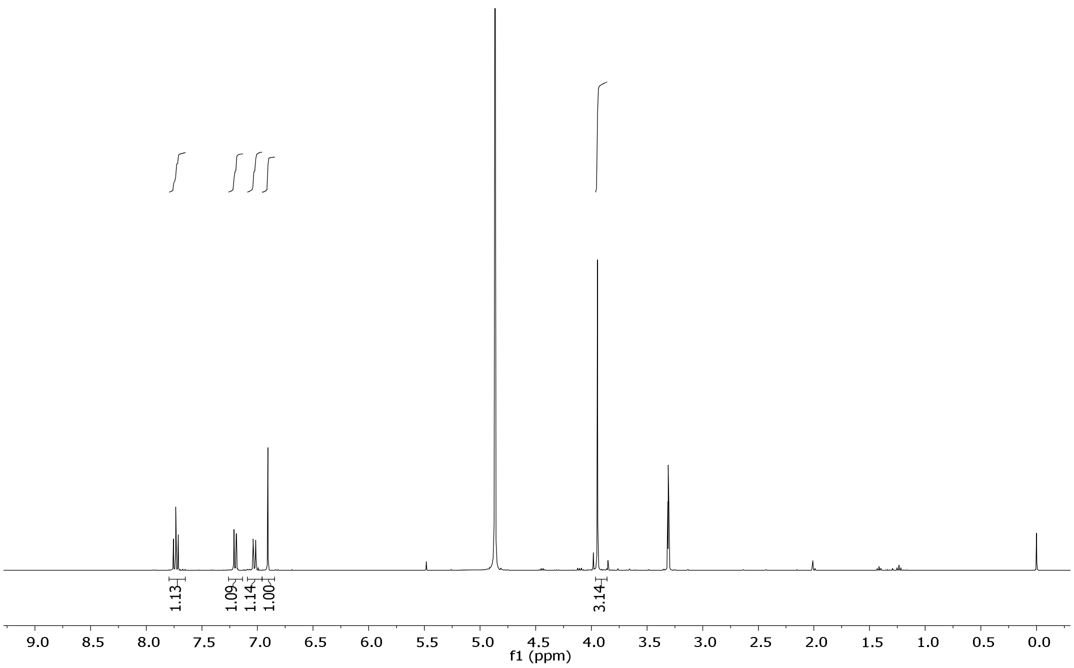

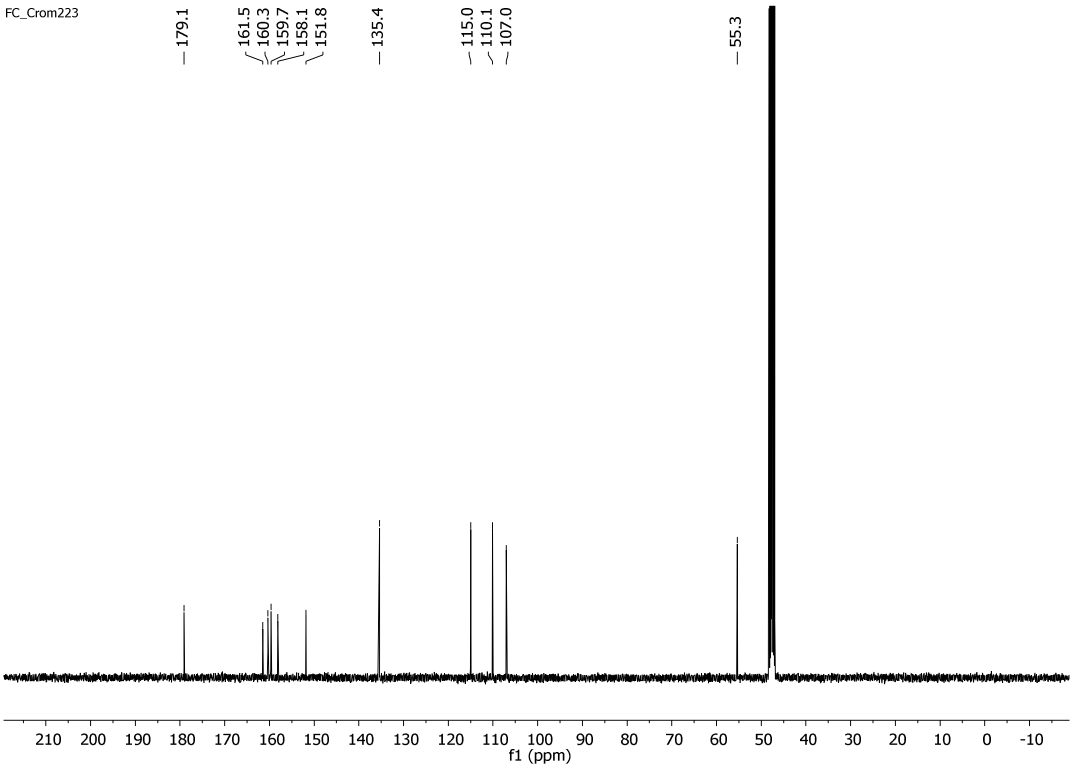

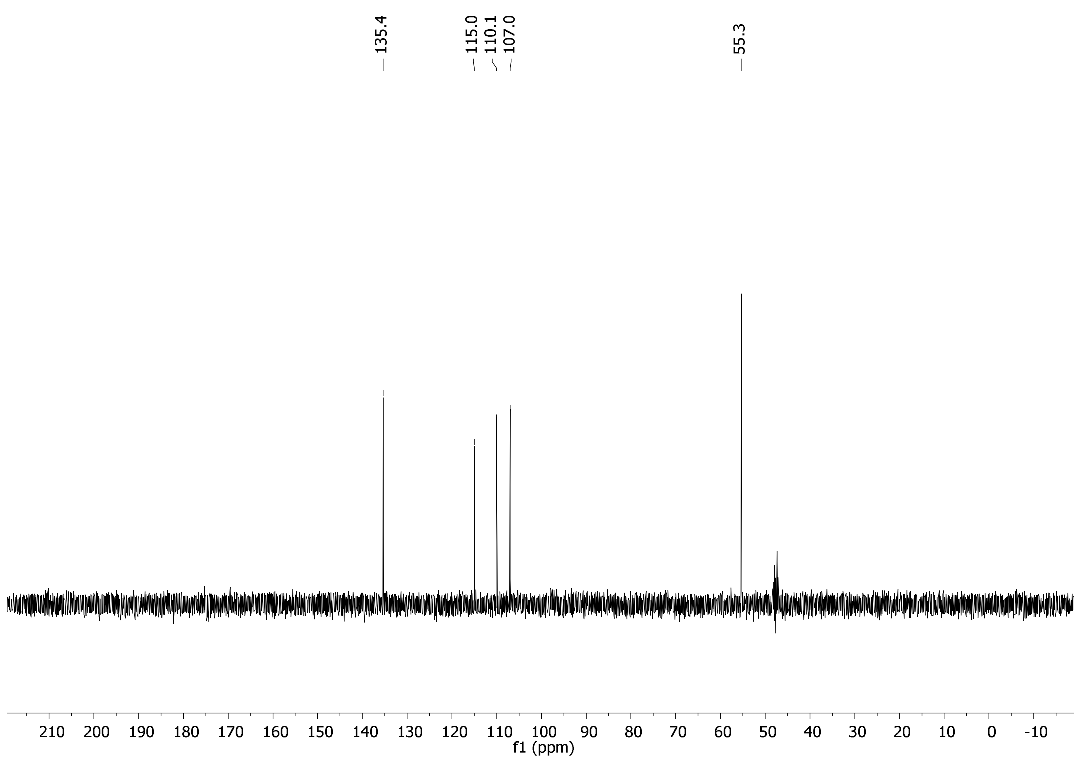

-7,8-Dimethoxy-4-oxo-4*H*-chromene-2-carboxylic acid (10B)

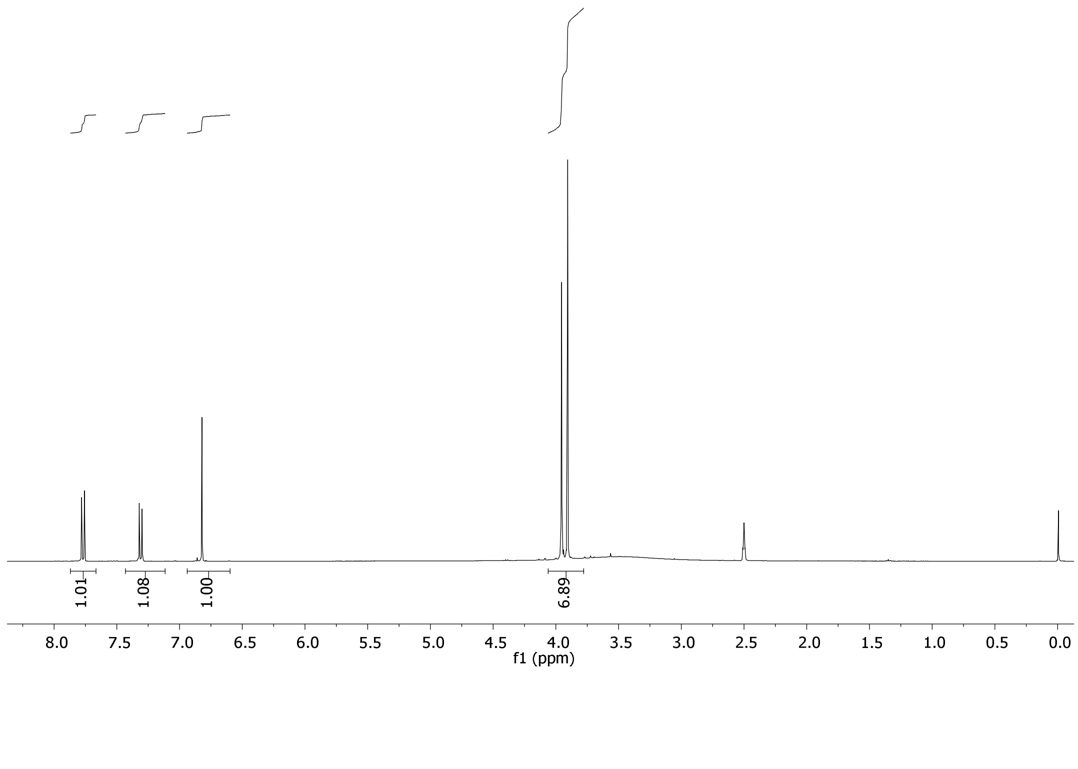

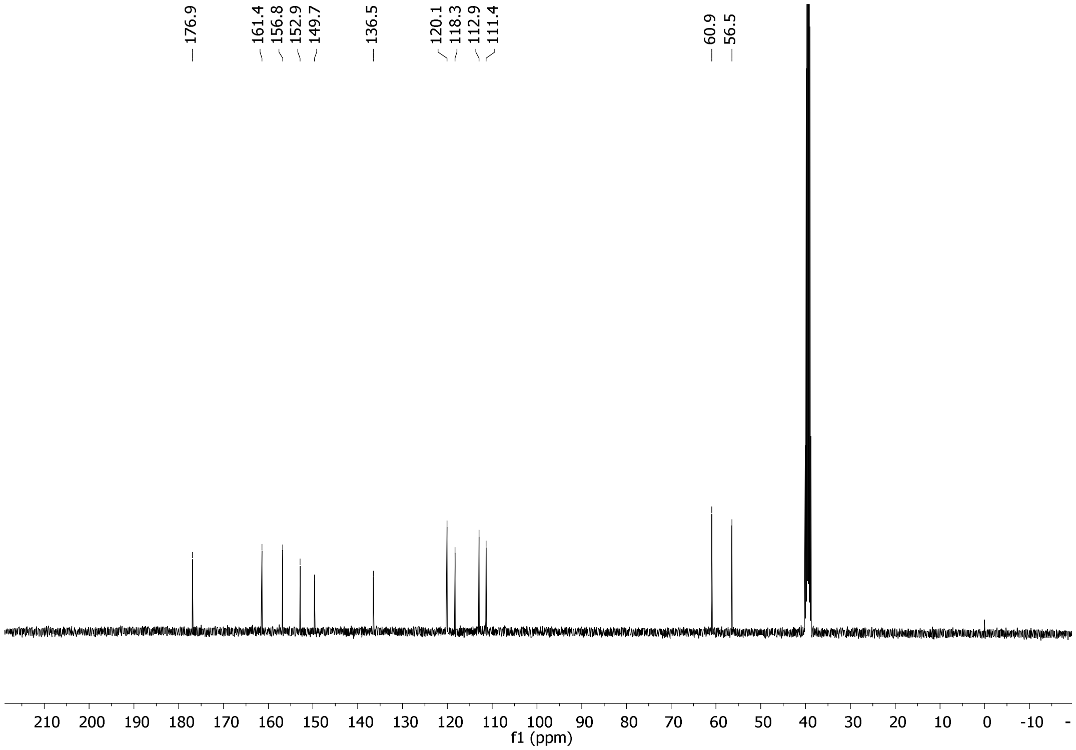
176.9
161.4
156.8
152.9
149.7
136.5
120.1
118.3
112.9
111.4
60.9
56.5
210 200 190 180 170 160 150 140 130 120 110 100 90 80 70 60 50 40 30 20 10 0 -10
f1 (ppm)

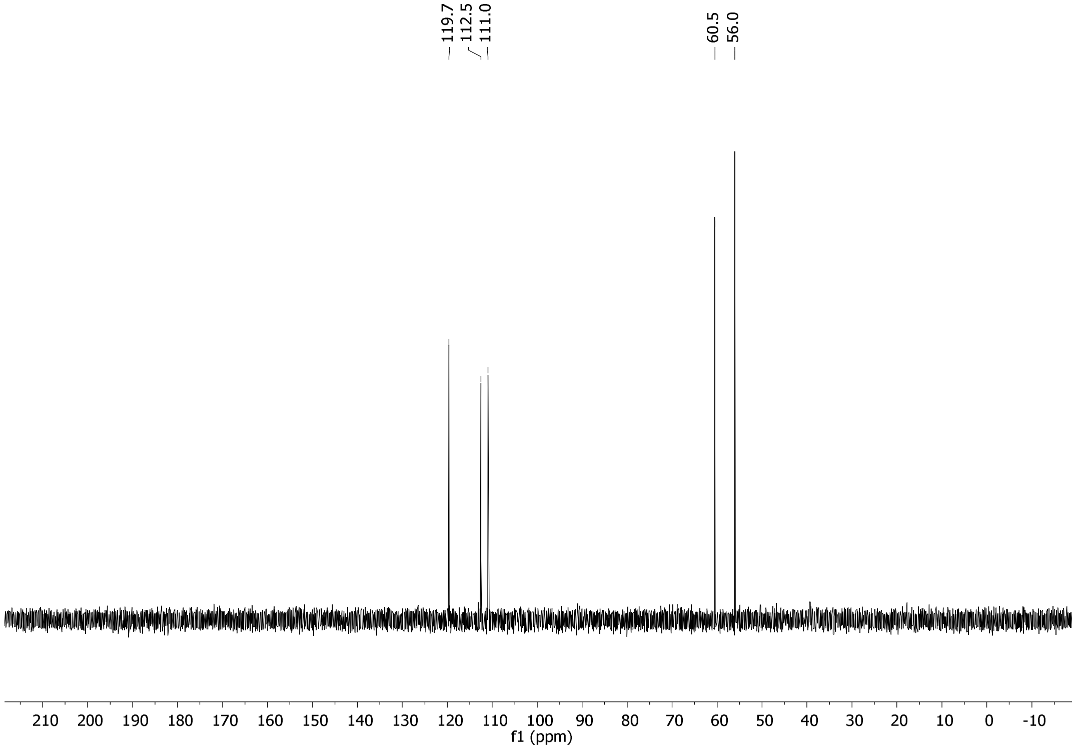
119.7
112.5
111.0
60.5
56.0
210 200 190 180 170 160 150 140 130 120 110 100 90 80 70 60 50 40 30 20 10 0 -10
f1 (ppm)

- **Methyl 6-nitro-4-oxo-4*H*-chromene-2-carboxylate (11B)**

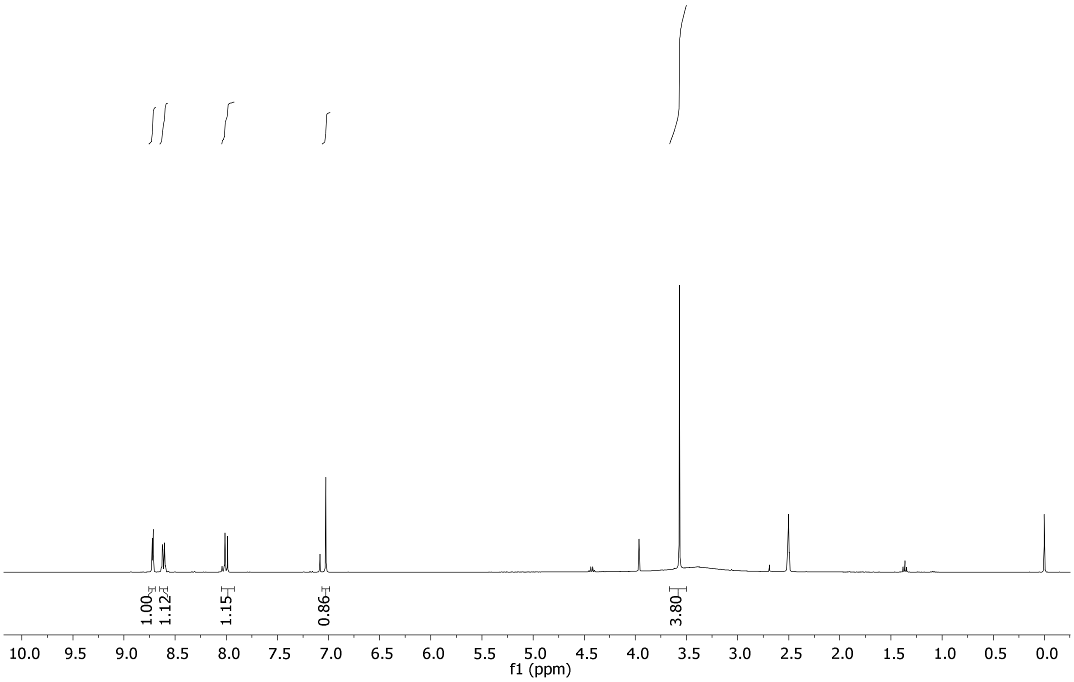

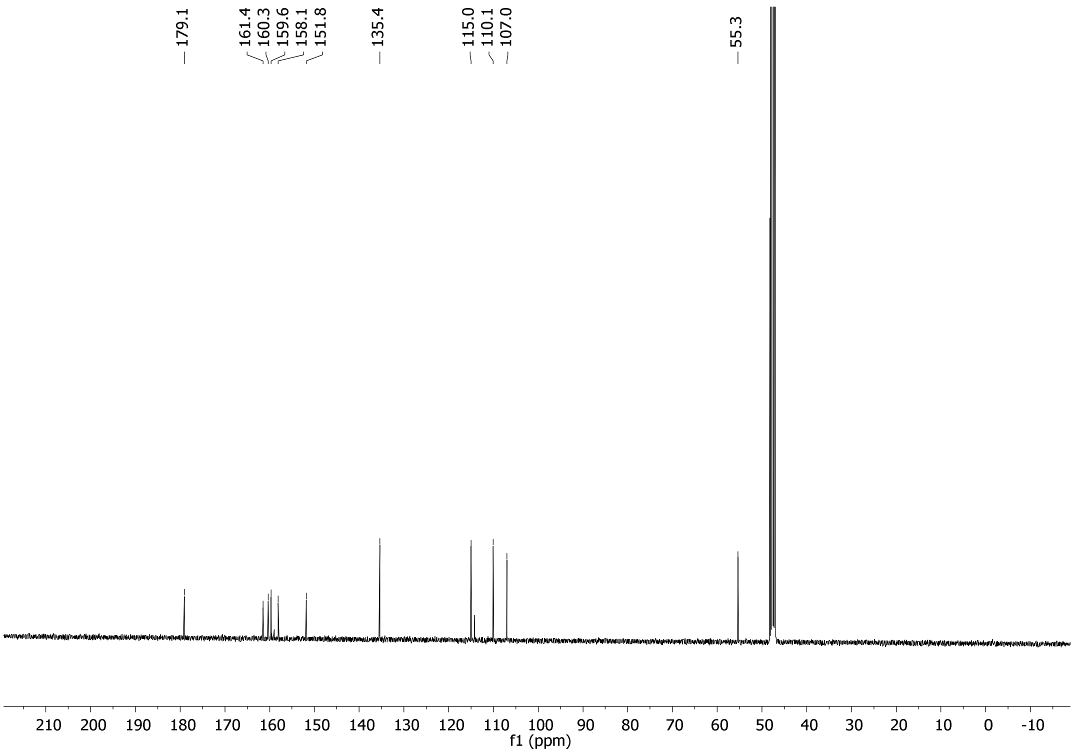

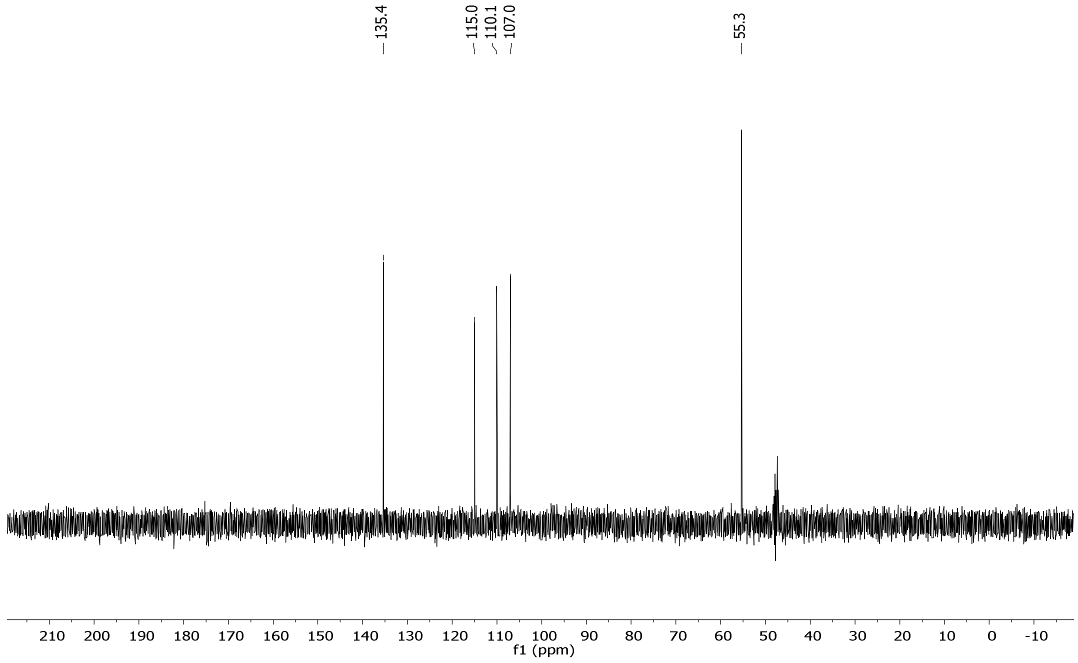

135.4
115.0
110.1
107.0
55.3
210 200 190 180 170 160 150 140 130 120 110 100 90 80 70 60 50 40 30 20 10 0 -10
f1 (ppm)
